# The Harbin Cohort Study on Diet, Nutrition and Chronic Non-Communicable Diseases: Study Design and Baseline Characteristics

**DOI:** 10.1371/journal.pone.0122598

**Published:** 2015-04-09

**Authors:** Lixin Na, Xiaoyan Wu, Rennan Feng, Jie Li, Tianshu Han, Liqun Lin, Li Lan, Chao Yang, Ying Li, Changhao Sun

**Affiliations:** 1 National Key Discipline, Department of Nutrition and Food Hygiene, School of Public Health, Harbin Medical University, Harbin, P. R. China; 2 Harbin Center for Disease Control and Prevention, Harbin, P. R. China; National University of Singapore, SINGAPORE

## Abstract

Diet and nutrition have been reported to be associated with many common chronic diseases and blood-based assessment would be vital to investigate the association and mechanism, however, blood-based prospective studies are limited. The Harbin Cohort Study on Diet, Nutrition and Chronic Non-communicable Diseases was set up in 2010. From 2010 to 2012, 9,734 participants completed the baseline survey, including demographic characteristics, dietary intake, lifestyles and physical condition, and anthropometrics. A re-survey on 490 randomly selected participants was done by using the same methods which were employed in the baseline survey. For all participants, the mean age was 50 years and 36% of them were men. Approximately 99.4 % of cohort members donated blood samples. The mean total energy intake was 2671.7 kcal/day in men and 2245.9 kcal/day in women, the mean body mass index was 25.7 kg/m^2^ in men and 24.6 kg/m^2^ in women, with 18.4% being obese (≥28 kg/m^2^), 12.7% being diabetic, and 29.5% being hypertensive. A good agreement was obtained for the physical measurements between the baseline survey and re-survey. The resources from the cohort and its fasting and postprandial blood samples collected both at baseline and in each follow-up will be valuable and powerful in investigating relationship between diet, nutrition and chronic diseases and discovering novel blood biomarkers and the metabolism of these biomarkers related to chronic diseases.

## Why Was the Cohort Set Up?

Chronic non-communicable diseases (NCDs), also known as chronic diseases, are the leading causes of death globally. The WHO data suggests that chronic diseases account for 60% of the world’s deaths, and that closes to 80% of these deaths occur in low- and middle-income countries [1–5]. According to national data, chronic diseases accounted for an estimated 80% of deaths and 70% of total disease burden in China in 2005[3]. Mortality rates from many common chronic diseases in most Western countries such as ischaemic heart disease have been decreasing, on the contrary, they are increasing in China as a result of adverse changes in lifestyle, diet and tobacco use [6–8]. As for each chronic disease, large unexplained variation between different populations still exists, which suggests that some non-genetic causes remain to be identified[9]. In addition, genetic factors and gene-environment interactions likely influence the risk of developing a human disease [10]. Consequently, large blood-based prospective cohort studies would be vital for the evaluation of the relevance of both genetic and environmental factors, and their interactions, as determinants for common chronic diseases [9].

Some important risk factors for chronic diseases, such as obesity, inactivity, excessive consumption of saturated fatty acids, and etc., are already known in a large number of human studies [11–15]. But most of the researches have been concentrated on Western populations and the results may not to be directly generalizable to other populations. Furthermore, dietary patterns are different in different populations. Therefore, studies on the effect of diet on chronic diseases in different populations are necessary for comprehensively and deeply understanding the effect of nutrition on health and the possible mechanisms. Several prospective studies on major chronic diseases in China have been conducted [3,9,16–21], however, they were limited by lack of blood samples or collecting blood samples only at baseline survey[3,9,16–21], or limited data collection on risk exposures and outcome measures especially for diet and nutrients [3,16,17]. To date, studies which were primarily designed to investigate the association between diet, nutrition and chronic diseases are rare, and prospective studies designed to collect blood samples not only at baseline but also in each follow-up are limited.

In 2010, we launched a large cohort study in Harbin, the capital and largest city of Heilongjiang province in China's northeast region, the Harbin Cohort Study on Diet, Nutrition and Chronic Non-communicable Diseases (HDNNCDS). The HDNNCDS was primarily designed to investigate the relationship between diet, nutrition and chronic diseases. In this paper, we report the detailed survey methods and the main baseline characteristics of the participants.

## Who Is in the Cohort?

### Ethics statement

The study protocol was approved by the Ethics Committee of the Harbin Medical University, and written informed consent was provided by all participants.

### Study population

The HDNNCDS covered 7 urban administrative regions of Harbin. Each region was divided into 3 strata according to their financial situation and a total of 42 communities were randomly selected from each stratum in each administrative region by employing a stratified multistage random cluster sampling design. Residents were eligible to participate in the study if they: 1) were between 20 and 74 years old, 2) have been living in Harbin for at least two years, 3) were without cancer or type 1 diabetes mellitus. Of the 12 865 eligible people, 9 828 subjects participated in the study, with a participation rate of 76.4 percent. The reasons for non-participation were uninterested in the study (4.6%), time constraints (7.8%), inability to understand the study (10.1), and no reason given (1.1%). Of those participants who completed the survey, 94 participants were later found to be younger than age 20 or older than age 74 years at the time of the baseline interview and thus were excluded from the cohort. Consequently, 9,734 subjects consisting of 3 526 men and 6 208 women constituted the cohort.

## What Has Been Measured?

### Baseline survey

A baseline survey was conducted in all study participants; detailed in-person interviews were administered by trained personnel using a structured questionnaire. The questionnaire included the following three sections: 1) demographic characteristics, 2) dietary habits, 3) lifestyles and physical condition.

The section of dietary habits was evaluated by the validated food frequency questionnaire (FFQ) containing the data regarding usual dietary intake over the past 12 months, including 103 food items from 14 food groups which are rice, wheaten food, potato starch and its products, beans and its products, vegetables, fruits, livestock and its products, poultry and its products, milk and its products, eggs and its products, fish and its products, snack, beverage, and ice cream. Information on favorite type of food with low, moderate, or high salt was also collected. Before the baseline survey, the following study was conducted to evaluate the validity and repeatability of the FFQ in 2009. A random subsample of 200 participants was recruited from 4 of the 42 study communities to complete two FFQs (FFQ1 and FFQ2) and a 3-day dietary record (DR). After adjusting for energy intake, major nutritional factors (staple food, poultry, fish, vegetable, fruit, beans, and milk products) which were assessed by the two FFQs and by FFQ2 and DRs correlated well. The correlation coefficients were 0.61–0.70 and 0.61–0.69, respectively. When the above major nutritional factors were categorized by their quartiles, the proportion of subjects that were classified into the same quartile from the FFQ2 and DR was 40%-49%. Misclassification of subjects into the opposite quartile was 2%-6%. This indicates that the FFQ used in the HDNNCDS is a reliable method for assessing dietary intake.

The section of lifestyles and physical condition mainly included prior disease history, family history of diseases, exercise, labor intensity, cigarette smoking, alcohol consumption, sleep habit, history of calcium deficiency and supplementation, and condition of taking medicines and health products in the past 12 months.

Anthropometric measurements, including height, weight, and waist circumferences (WC), were also taken at baseline by well-trained examiners, with participants wearing light, thin clothing and no shoes. Body weight and height were measured to the nearest 0.1 kg and 0.1 cm, respectively. Blood pressures were measured 3 times with a standard mercury sphygmomanometer on the right arm of each subject after a 10-minute rest in a sitting position, and the mean values were used for analysis. Body mass index (BMI, kg/m^2^) was calculated as weight (kg) divided by the square of the height in meters (m^2^). Fat mass (FM) was measured by using the electric impedance method with a body FM analyzer (OMRON HBF-306, Omron Corporation, Dalian, China).

Of the 9,734 study participants, 9 679 subjects (99.4 percent) donated blood samples at baseline, including fasting and postprandial (2 hours after drinking a 75 grams glucose-containing water) blood samples. After collection, plasma samples were kept in a portable, insulated bag with ice packs (at about 0–4°C) and were processed within 6 hours for long-term storage at -80°C. The blood sample repositories for the study are equipped with appropriate alarm systems and emergency electricity backup to prevent accidental thawing. Except for the measurements of blood glucose and lipid profile, the metabolomics analysis of plasma was additionally performed on ultra-performance liquid chromatography/mass spectrometry (UPLC/MS) and nuclear magnetic resonance (NMR) to select the potential metabolites related to chronic diseases.

Current smokers were defined as those who have smoked at least 100 cigarettes lifetime and smoke every day or some days now. Current drinkers were defined as those who consumed≥1 alcoholic drink each month in the past 12 months before the survey. Regular exercise was defined as any kind of recreational or sport physical activity other than walking for work or life performed at least 30 minutes for three or more days per week.

### Outcomes ascertainment

Information on previous chronic diseases, such as diabetes, hypertension, hyperlipedimia, diseases of the hematopoietic system, chronic obstructive pulmonary disease (COPD), and asthma, was collected in the baseline survey by asking questions like: “Whether or not it has been diagnosed?”, “When have you been diagnosed?”, “Did you control the disease?”, and etc. In addition, some chronic diseases were diagnosed by combining information collected in the questionnaire with the measurements of blood samples or blood pressures, for example, diabetes was defined as fasting blood glucose≥7.0 mmol/L, and/or 2-h glucose≥11.1 mmol/L, and/or taking medications for diabetes, and hypertension was defined as systolic blood pressure≥140 mmHg or diastolic blood pressure≥90 mmHg, and/or taking medications for hypertension.

### Quality control procedures and data entry/editing

All surveys at baseline were recorded in-person by the well trained interviewers and the questionnaire did not include any self-administered information which was needed to be filled in by participants themselves. A total of 43 interviewers participated in the field work at baseline survey and sample collection, and 36 interviewers completed 98.6 percent of the interviews. Double entry of the questionnaires was done by two different clerks. Records that were inconsistent were manually checked and corrected. Computer programs were also created to check the logic and reasonable range of responses throughout the questionnaire to identify contradictory responses.

### Re-survey

A re-survey was conducted between January 2013 and April 2013 following completion of the baseline survey. About 5% participants (490 participants: 153 men and 337 women) were randomly chosen from all participants and completed the re-survey. The survey procedures and data collection were much the same as those used in the baseline survey except for a few additional questions (e.g. recent hospitalization).

## What Will Be Done in the Cohort Follow-Up?

The data collection of follow-ups will be conducted periodically till death. Each living participant will be visited by an in-person interview by collecting information much the same as those at baseline survey and health history. Common chronic diseases were recorded that have occurred since the last in-person contact. Any deaths information among cohort members will be gathered through the record linkage which covered near 100% mortality of our study participants by Harbin Center for Disease Control and Prevention. The consistency of survey components will be evaluated based on the same information collected in each follow-up of the future. New and more detailed data collection to the study will be considered for the follow-up surveys to calibrate and enhance the information collected at the baseline survey. For example, physical activity or dietary assessment would be enhanced based on the last follow-up survey. In addition to the above information, the blood samples will be collected from all living participants in each follow-up and stored by using the same method as that at baseline until analysis.

## What Statistical Analysis Has Been Performed on the Baseline Data?

The mean values and prevalence were calculated separately for men and women for selected baseline variables, and age-standardized prevalence of prior chronic diseases by 2010 Chinese national survey for Heilongjiang province were also calculated. Correlations between baseline and re-survey data were estimated by Spearman correlation coefficients. All data were analyzed by using SAS (version 9.2; SAS Institute, Inc., Cary, NC).

## What Main Characteristics Have Been Analyzed at Baseline?

**Fig 1:**
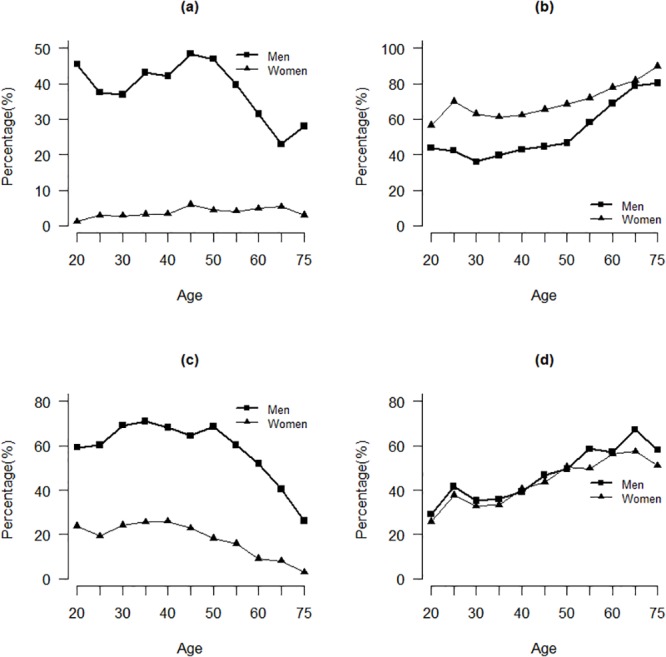
Prevalence of selected baseline variables by sex and by age. (a) percentage current smokers; (b) percentage passive smokers; (c) percentage current drinkers; (d) percentage regular exercisers.

Selected characteristics of study participants at baseline are presented in Table 1. Of the 9,734 participants in this cohort study, the mean age was 50 years and 36% of them were men, and more than 80% of the participants had attended at least middle school. Premenopausal women constituted nearly 56% of the women participants. The percentage of current smokers was much higher in men than that in women (41.5 vs 4.2%). Among women, the prevalence of current smoking was uncommon and varied little by age (Fig 1(a)); among men, although the prevalence decreased if men were aged 50 years or older, it was still higher than 20%. The percentage of women suffered from passive smoke was higher than that of men (68.4 vs 50.3%), and the prevalence varied little for women but increased if men were older than 50 years old (Fig 1(b)). Large difference was also found between men and women for the current alcohol drinking (62.8 vs 19.1%), and the prevalence decreased if they aged 50 years or older either for men or for women (Fig 1(c)). For the regular exercise, the prevalence in men was a little higher than that in women (48.2 vs 45.5%), but there has been an increase either for men or men when they were getting older (Fig 1(d)). In addition, intake levels of total energy and major food groups are presented with mean and its standard deviation in Table 1. Women had lower intakes of total energy, staple food, poultry, fish, and beans, but higher intakes of fruit, and milk and its products compared with men.

**Table 1:** Selected characteristics of participants at baseline in the Harbin Cohort Study on Diet, Nutrition and Chronic Non-communicable Diseases (2010–2012), China.

Baseline characteristic^a^	Total (*n* = 9,734)	Men (*n* = 3,526)	Women (*n* = 6,208)
**Age group (years) (%)**			
20–30	3.5	4.1	3.1
31–40	15.5	17.1	14.6
41–50	30.1	30.1	30.0
51–60	34.7	32.4	36.1
61–74	16.2	16.3	16.2
Age (years, Mean (SD))	50.1(10.3)	49.7(10.5)	50.4(10.2)
**Education (%)**			
No formal education	1.8	1.1	2.2
Elementary school	4.8	3.2	5.7
Middle school	23.2	22.5	23.6
High school/secondary technical school	32.2	30.1	33.4
Technical school/college	31.2	36.0	28.6
Postgraduate degree or above	0.7	1.0	0.5
Unknown	6.1	6.2	6.0
**Lifestyle factors (%)**			
Current smoker	17.7	41.5	4.2
Passive smoke	61.6	50.3	68.4
Current alcohol drinker	34.7	62.8	19.1
Exercised regularly	46.4	48.2	45.5
Fond of salty food	20.4	26.5	16.9
**Dietary nutritional factors (mean(SD))**			
Energy intake (kcal/day)	2400.2(929.9)	2671.7(907.2)	2245.9(907.2)
Staple food(g/day)	361.5(184.3)	427.4(177.5)	324.1(177.5)
Poultry (g/day)	31.9(56.8)	37.6(56.6)	28.6(56.6)
Fish (g/day)	37.6(84.3)	42.7(84.2)	34.6(84.2)
Vegetable (g/day)	274.4(226.2)	271.7(226.2)	275.9(226.2)
Fruit (g/day)	151.2(159.6)	121.1 (158.0)	168.3(158.0)
Beans (g/day)	54.0(68.4)	60.6(68.2)	50.3(68.2)
Milk and its products (g/day)	90.6 (105.8)	79.7(105.5)	96.8(105.5)
**Menopausal status (%)**			
Premenopause			55.8
Postmenopause			34.9
Unknown			9.2

Abbreviation: SD, standard deviation.

^a^ Adjusted for age.

**Fig 2:**
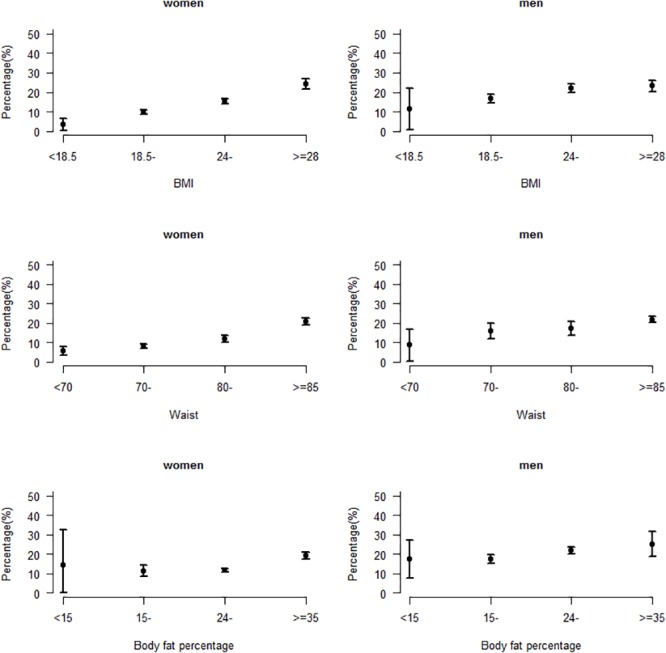
Associations of different measures of adiposity with diabetes at baseline. Prevalence of diabetes vs (a) BMI (kg/m^2^), (b) waist (cm), and (c) body fat percentage after adjustment for age.

Table 2 shows the anthropometric characteristics, prior disease history, and lipid profile of participants at baseline. Mean BMI was 25.7 kg/m^2^ in men and 24.6 kg/m^2^ in women, with approximate 18.4% being obese (BMI≥28 kg/m^2^) [22]. Age-standardized prevalence of common chronic diseases at baseline included diabetes (12.7%), hypertension (29.5%), hyperlipemia (18.9%), diseases of hematopoietic system (0.4%), COPD (2.8%), and asthma (1.0%). Compared with men, women had lower prevalence of diabetes, hypertension, and hyperlipemia. Among the measures of adiposity in the study, BMI and waist circumference were found to be more strongly associated with diabetes compared with body fat percentage adjusted for age at baseline in both men and women (Fig 2).

**Table 2:** Anthropometric characteristics, prior disease history, and lipid profile at baseline in the Harbin Cohort Study on Diet, Nutrition and Chronic Non-communicable Diseases (2010–2012), China.

Characteristics	Total (*n* = 9,734)	Men (*n* = 3,526)	Women (*n* = 6,208)
**Height (cm)** ^a^			
<155	14.6	0.8	22.3
155–159	20.8	3.0	30.9
160–164	23.7	10.9	31.1
≥165	40.9	85.3	15.7
Mean (SD)	163.2(8.1)	170.8(5.7)	158.9(5.7)
**BMI (kg/m^2^)** ^a^			
<18.5	1.7	1.0	2.1
18.5 to < 24	41.8	32.5	47.4
24.0 to < 28	38.2	42.9	35.4
≥28	18.4	23.6	15.2
Mean (SD)	25.0(3.5)	25.7(3.5)	24.6(3.5)
**Waist circumference(cm)** ^a^			
<70	4.8	1.3	6.9
70–79	22.2	9.6	29.5
80–84	19.1	13.3	22.4
≥85	53.9	75.7	41.2
Mean (SD)	86.0(10.3)	91.2(9.5)	83.0(9.5)
**Body fat percentage** ^a^			
<15	0.8	1.6	0.2
15–24	16.4	30.7	8.3
25–34	59.9	62.9	58.4
≥35	23.1	4.8	33.1
Mean (SD)	30.5(5.7)	26.9(5.0)	32.5(5.0)
**Prior disease history(%)**			
Diabetes			
Crude	16.6	20.6	14.4
Age-standardized^b^	12.7	16.1	11.0
Hypertension			
Crude	39.1	47.3	34.2
Age-standardized^b^	29.5	37.2	25.1
Hyperlipemia			
Crude	23.9	28.3	21.3
Age-standardized^b^	18.9	24.0	15.9
Diseases of the hematopoietic system			
Crude	0.5	0.5	0.6
Age-standardized^b^	0.4	0.4	0.5
Chronic obstructive pulmonary disease			
Crude	3.8	4.1	3.6
Age-standardized^b^	2.8	3.0	2.7
Asthma			
Crude	1.3	1.6	1.2
Age-standardized^b^	1.0	1.1	0.9
**Lipid profile (mean(SD))** ^a^			
TG (mmol/L)	1.8(1.9)	2.3(1.8)	1.5(1.8)
TCHO (mmol/L)	5.2(1.0)	5.1(1.0)	5.2(1.0)
HDL (mmol/L)	1.3(0.3)	1.1(0.3)	1.3(0.3)
LDL (mmol/L)	3.0(0.9)	3.0(0.9)	3.1(0.9)

Abbreviation: SD, standard deviation; BMI, body mass index; TG, triglyceride; TCHO, total cholesterol; HDL, high-density lipoprotein; LDL, low-density lipoprotein.

^a^ Adjusted for age.

^b^Age-standardized prevalence by 2010 Chinese national survey for Heilongjiang province.

A good agreement was obtained between the baseline and resurvey measurements for most of the variables, particularly for various physical measurements (Table 3). The height and weight were extremely highly correlated and the correlation coefficients were 0.98 and 0.96, respectively, whereas for waist circumference and body fat percentage, the correlation coefficients were 0.87 and 0.82, respectively. For systolic blood pressure and diastolic blood pressure, the correlation coefficients were 0.65 and 0.66, respectively.

**Table 3:** Spearman correlation coefficients for selected physical measurements between baseline survey and re-survey among 490 participants.

Variables	Total (*n* = 490)	Men (*n* = 153)	Women (*n* = 337)
Height	0.98	0.99	0.97
Weight	0.96	0.94	0.96
Waist circumference	0.87	0.83	0.84
Body fat percentage	0.82	0.80	0.83
SBP	0.65	0.73	0.71
DBP	0.66	0.68	0.64

Abbreviation: SBP, systolic blood pressure; DBP: diastolic blood pressure.

## What Are the Main Strengths and Weaknesses?

As a national key discipline, the support from our nation enables us to successfully construct the HDNNCDS, a prospective follow-up investigation in a cohort of adult men and women in China. It was primarily designed to investigate the association between diet, nutrition and chronic diseases.

The HDNNCDS was conducted in Harbin, which is the capital and largest city of Heilongjiang province in China's northeast region, as well as is the tenth most populous city nationally. Due to the Siberian high and its location above 45 degrees north latitude, the city is known for its coldest weather and longest winter among major Chinese cities. The dietary pattern in Harbin is characterized by ingestion of high calorie during winter. And fewer kinds of vegetables and fruits can be planted under this climate condition, so the supply of vegetables and fruits is relatively lack in winter. Compared with other studies conducted in south area in China, the study participants in HDNNCDS have higher intake of total energy, but lower intakes of vegetables and fruits either in men or women [19,23,24]. In addition, vitamin D deficiency is very common in this area due to the poor sunlight [25]. On the other hand, the incidence of chronic diseases has been reported to be higher in north region than that in south region in China [26]. As shown in our study, the prevalence of diabetes and hypertension were a little bit higher than or comparable to those reported in other studies in China [2,27]. The age-standardized prevalence of diabetes in our study for overall participants, women, and men were 12.7%, 16.1%, and 11.0%, respectively, whereas those reported by Xu et al. were 11.6%, 12.1%, and 11.0%, respectively. The age-standardized prevalence of hypertension in our study for overall participants, women, and men were 29.5%, 37.2%, and 25.1%, respectively, whereas those reported by Gao et al. were 26.6%, 29.2%, and 24.1%, respectively. Therefore, the HDNNCDS, a representative cohort with high prevalence of chronic diseases, would be useful to investigate the association between diet, nutrition and chronic diseases. Meanwhile, the results from the HDNNCDS might be valuable to the prevention and treatment of chronic diseases for other populations who live in the area with the similar high latitude degrees worldwide.

Some cohort studies obtained baseline information mainly by a self-administered mail survey [28], which may affect the quality of data by educational level and result in potential bias. To enhance the quality of data in the present study, in-person interviews were used to collect data both at baseline and in each follow-up. In addition to the detailed data collection of dietary intake, an extensive range of data was collected at baseline. For example, different measures of adiposity in the study will allow for reliable assessment of their relative values for different conditions. BMI and waist circumference were found to be more strongly associated with diabetes compared with body fat percentage in the present study, but this needs to be confirmed in prospective incidence data.

The blood samples were collected at baseline and will be collected from all living participants in each follow-up in the future. The nutrition status of the population can be comprehensively assessed with the combination of dietary questionnaire and blood analysis. The current number of 9,734 in the cohort is compatible and optimal for us to achieve extensive and precise blood analysis. In addition, the collection of both fasting and postprandial blood samples (2 hours after drinking a 75 grams glucose-containing water) at baseline and each follow-up will substantially enhance and expand our assessment of dietary nutrition, metabolism and its effect on health. By analyzing the postprandial blood samples, we not only can comprehensively study chronic diseases, such as diabetes, but also can investigate the postprandial metabolic changes of some nutrients (e.g. glucose, lipid). It is well known that the body is in the postprandial state for most of the day. Changes in nutrients and metabolism in the postprandial state could contribute to the alteration of the pathophysiological function of the body; and several studies have reported that change in postprandial index in blood is related to certain diseases[29]. Therefore, investigation of the potential effect of change in the nutrient metabolism profile in the postprandial state as well as the fasting state in this study can provide deep insight into the study of relationship between diet, nutrition and chronic diseases.

Noteworthy, metabolomics is applied in the study. Metabolomics is an emerging science dedicated to the global study of small molecule metabolites; their entire composition, dynamics, and responses to disease or environmental changes in cells, tissues, and biofluids[30,31]. To date, metabolomics has been successfully utilized in disease diagnosis [32], biomarker screening [33], characterization of biological pathways[34], and nutrition research. UPLC/MS and NMR have been well established in metabolomics. UPLC/MS can detect early subtle perturbations of metabolism in metabolomics study for its high sensitivity, resolution, and rapid separation. NMR provides high reproducibility and is a powerful tool in terms of quantification [35,36]. The results from these two techniques for the same data could complement and reinforce each other. We have both UPLC/MS and NMR in our lab, which enables us to research metabolomics comprehensively. In addition, our group has successfully applied UPLC/MS metabonomic platform in pathogenesis elucidation and biomarker discovery in various diseases [36–41] and investigations based on NMR metabolomics has been performed. In this study, we focused on identifying potential biomarkers related to malnutrition as well as chronic diseases, and metabolic clues to link between nutrition and diseases. Following the completion of baseline survey, plasma metabolomic analysis on UPLC/MS and NMR has been conducted.

The limitations of this study include that (i) a low percentage of male participants in the cohort, which was mainly resulted from the higher non-participation rate in men (18.9%) than that in women (4.7%), and thus, age and sex standardized or age standardized characteristics in men and women should be considered in the analyses; (ii) relatively limited demographic information at baseline, which needs to be enhanced in the follow-up surveys, such as marriage, nationality, and etc.

In summary, we have successfully established a population-based representative cohort of 9,734 adults for investigating relationship between diet, nutrition and chronic diseases in alpine region. The fasting and postprandial blood samples collected not only at baseline but also in each follow-up will be a unique and powerful resource for investigating the association between diet, nutrition metabolism and chronic diseases and discovering novel metabolic biomarkers.

## Supporting Information

S1 Questionnaire The questionnaire of the Harbin Cohort Study on Diet, Nutrition and Chronic Non-communicable Diseases (HDNNCDS). (DOCX)
