# Supplementary material for: The Harbin Cohort Study on Diet, Nutrition and Chronic Non-Communicable Diseases: Study Design and Baseline Characteristics
Source: PLoS One. 2015 Apr 9;10(4):e0122598. doi: 10.1371/journal.pone.0122598 (PMC4391912; doi:10.1371/journal.pone.0122598)
Supplement: S1 Questionnaire — (DOCX) [file pone.0122598.s001.docx]

|  |  | CODE1 (Personal ID):_______________ |
| --- | --- | --- |
|  |  | Date: ________year ____ month____ day |
|  |  |  |

# Harbin Cohort Study on Diet, Nutrition and Chronic Non-communicable Diseases

# Interview Questionnaire

# (english translation)

Chronic non-communicable diseases, also known as chronic diseases, are a major global health problem. According to national data, chronic diseases accounted for an estimated 80% of deaths and 70% of total disease burden in China in 2005. At present, our knowledge between the diet, nutrition and chronic disease is still limited in China. In our daily lives, we might have contacted with some factors that are considered relevant to chronic diseases. The study of these dietary factors will play an import role in preventing these chronic diseases.

The department of nutrition and food hygiene, which is the National Key Discipline in the Public Health School of Harbin Medical University, is going to conduct a survey on adults’ health in urban Harbin. Based on the agreement of your committee, we invite all adults aged 20-74 years old without malignant tumors or type 1 diabetes in the committee to participate in this survey. Your participation would be really appreciated and contribute to prevention and treatment of chronic diseases.

Interviewer, please fill the following column before you complete the interview:

Has there been a blood sample? 1 …… yes 2 …… no | __ |

National Key Discipline

Department of Nutrition and Food Hygiene

Public Health School

Harbin Medical University

**Part ONE dEMOGRAPHIC CHRACTERISTICS**

1. Name: ________________________

2.Telephone: _____________________

3.Address:________________________

4. Sex: ①male

②female

(Are you menopausal?

①yes (what was the date of your last period? ____ year __month) ②no）

5. What was your date of birth? (Do not use lunar calendar) ________ year _____ month _____ day

6. What is your education level?

①No formal education ②Elementary school

③Junior high school ④High school/ secondary technical school

⑤Technical school/college ⑥Postgraduate degree or above

⑦Unknown

**PART TWO DIETARY HABITS**

1. In the past year, how many people in your family, including yourself, have ate together? _______ persons.

2. In the past year, how much did your family consume per month (50 g):

vegetable oil: _______(50 g)

soy bean oil: _______ (50 g)

peanut oil: _______ (50 g)

lard: _______ (50 g)

salt: _______ (50 g)

3. In the past year, which type of food did you prefer?

①with low salt ②with moderate salt ③with high salt

4. Dietary habit

Now please recall the following dietary habit in the past year. Please note: you should tell me the amount of the specific food you consumed by yourself, not your entire family. Please tell me whether you ate the food I read and frequency and its amount.

For example, I will ask you if you ate rice every day, every week, every month, every year, or not at all. If you ate rice every day, you tell that: “I ate rice every day.” I will then ask you how much you normally ate at a given unit of time (Amt. consumed (50g)).

（1）Rice

| Names of food | Frequency of food consumption | | | | | Amt. consumed  (50 g) |
| --- | --- | --- | --- | --- | --- | --- |
| 1. rice | ①Every day  ______ | ②Every week  ______ | ③Every Month  _____ | ④Every Year  _____ | ⑤Not at all  _____ |  |

(2) Wheaten food

| Names of food | Frequency of food consumption | | | | | Amt. consumed  (50 g) | Note |
| --- | --- | --- | --- | --- | --- | --- | --- |
| 2. foxtail millet | ①Every day  ______ | ②Every week  ______ | ③Every Month  _____ | ④Every Year  _____ | ⑤Not at all  _____ |  |  |
| 3. maize | ①Every day  ______ | ②Every week  ______ | ③Every Month  _____ | ④Every Year  _____ | ⑤Not at all  _____ |  |  |
| 4. noodle | ①Every day  ______ | ②Every week  ______ | ③Every Month  _____ | ④Every Year  _____ | ⑤Not at all  _____ |  |  |
| 5. steamed twisted roll and steamed bread | ①Every day  ______ | ②Every week  ______ | ③Every Month  _____ | ④Every Year  _____ | ⑤Not at all  _____ |  |  |
| 6. bread | ①Every day  ______ | ②Every week  ______ | ③Every Month  _____ | ④Every Year  _____ | ⑤Not at all  _____ |  |  |
| 7. others  (please give the ‘name of the food ’in the ‘Note’ column) | ①Every day  ______ | ②Every week  ______ | ③Every Month  _____ | ④Every Year  _____ | ⑤Not at all  _____ |  |  |

(3) Potato starch and its products

| Names of food | Frequency of food consumption | | | | | Amt. consumed  (50 g) |
| --- | --- | --- | --- | --- | --- | --- |
| 8. potato | ①Every day  ______ | ②Every week  ______ | ③Every Month  _____ | ④Every Year  _____ | ⑤Not at all  _____ |  |
| 9. sweet potato | ①Every day  ______ | ②Every week  ______ | ③Every Month  _____ | ④Every Year  _____ | ⑤Not at all  _____ |  |
| 10.vermicelli | ①Every day  ______ | ②Every week  ______ | ③Every Month  _____ | ④Every Year  _____ | ⑤Not at all  _____ |  |

(4) Beans and its products

| Names of food | Frequency of food consumption | | | | | Amt. consumed  (50 g) |
| --- | --- | --- | --- | --- | --- | --- |
| 11. Tofu | ①Every day  ______ | ②Every week  ______ | ③Every Month  _____ | ④Every Year  _____ | ⑤Not at all  _____ |  |
| 12. dried bean curd | ①Every day  ______ | ②Every week  ______ | ③Every Month  _____ | ④Every Year  _____ | ⑤Not at all  _____ |  |
| 13. soybean milk | ①Every day  ______ | ②Every week  ______ | ③Every Month  _____ | ④Every Year  _____ | ⑤Not at all  _____ |  |

(5) Vegetables

| Names of food | Frequency of food consumption | | | | | Amt. consumed  (50 g) |
| --- | --- | --- | --- | --- | --- | --- |
| 14. Mooli | ①Every day  ______ | ②Every week  ______ | ③Every Month  _____ | ④Every Year  _____ | ⑤Not at all  _____ |  |
| 15.garden radish | ①Every day  ______ | ②Every week  ______ | ③Every Month  _____ | ④Every Year  _____ | ⑤Not at all  _____ |  |
| 16.carrot | ①Every day  ______ | ②Every week  ______ | ③Every Month  _____ | ④Every Year  _____ | ⑤Not at all  _____ |  |
| 17. spinach | ①Every day  ______ | ②Every week  ______ | ③Every Month  _____ | ④Every Year  _____ | ⑤Not at all  _____ |  |
| 18. soybean sprouts | ①Every day  ______ | ②Every week  ______ | ③Every Month  _____ | ④Every Year  _____ | ⑤Not at all  _____ |  |
| 19. sprouts of mung bean | ①Every day  ______ | ②Every week  ______ | ③Every Month  _____ | ④Every Year  _____ | ⑤Not at all  _____ |  |
| 20. eggplant | ①Every day  ______ | ②Every week  ______ | ③Every Month  _____ | ④Every Year  _____ | ⑤Not at all  _____ |  |
| 21. tomato | ①Every day  ______ | ②Every week  ______ | ③Every Month  _____ | ④Every Year  _____ | ⑤Not at all  _____ |  |
| 22. chili green | ①Every day  ______ | ②Every week  ______ | ③Every Month  _____ | ④Every Year  _____ | ⑤Not at all  _____ |  |
| 23. white gourd | ①Every day  ______ | ②Every week  ______ | ③Every Month  _____ | ④Every Year  _____ | ⑤Not at all  _____ |  |
| 24. cucumber | ①Every day  ______ | ②Every week  ______ | ③Every Month  _____ | ④Every Year  _____ | ⑤Not at all  _____ |  |
| 25. pumpkin | ①Every day  ______ | ②Every week  ______ | ③Every Month  _____ | ④Every Year  _____ | ⑤Not at all  _____ |  |
| 26. cocozelle | ①Every day  ______ | ②Every week  ______ | ③Every Month  _____ | ④Every Year  _____ | ⑤Not at all  _____ |  |
| 27. garlic bolt | ①Every day  ______ | ②Every week  ______ | ③Every Month  _____ | ④Every Year  _____ | ⑤Not at all  _____ |  |
| 28. garlic sprout | ①Every day  ______ | ②Every week  ______ | ③Every Month  _____ | ④Every Year  _____ | ⑤Not at all  _____ |  |
| 29. allium fistulosum | ①Every day  ______ | ②Every week  ______ | ③Every Month  _____ | ④Every Year  _____ | ⑤Not at all  _____ |  |
| 30. onions | ①Every day  ______ | ②Every week  ______ | ③Every Month  _____ | ④Every Year  _____ | ⑤Not at all  _____ |  |
| 31. Chinese chives | ①Every day  ______ | ②Every week  ______ | ③Every Month  _____ | ④Every Year  _____ | ⑤Not at all  _____ |  |
| 32. Chinese cabbage | ①Every day  ______ | ②Every week  ______ | ③Every Month  _____ | ④Every Year  _____ | ⑤Not at all  _____ |  |
| 33. sauerkraut | ①Every day  ______ | ②Every week  ______ | ③Every Month  _____ | ④Every Year  _____ | ⑤Not at all  _____ |  |
| 34. rape | ①Every day  ______ | ②Every week  ______ | ③Every Month  _____ | ④Every Year  _____ | ⑤Not at all  _____ |  |
| 35. flowering Chinese cabbage | ①Every day  ______ | ②Every week  ______ | ③Every Month  _____ | ④Every Year  _____ | ⑤Not at all  _____ |  |
| 36. cabbage | ①Every day  ______ | ②Every week  ______ | ③Every Month  _____ | ④Every Year  _____ | ⑤Not at all  _____ |  |
| 37. red cabbage | ①Every day  ______ | ②Every week  ______ | ③Every Month  _____ | ④Every Year  _____ | ⑤Not at all  _____ |  |
| 38. cauliflower | ①Every day  ______ | ②Every week  ______ | ③Every Month  _____ | ④Every Year  _____ | ⑤Not at all  _____ |  |
| 39. broccoli | ①Every day  ______ | ②Every week  ______ | ③Every Month  _____ | ④Every Year  _____ | ⑤Not at all  _____ |  |
| 40. cabbage mustard | ①Every day  ______ | ②Every week  ______ | ③Every Month  _____ | ④Every Year  _____ | ⑤Not at all  _____ |  |
| 41. celery | ①Every day  ______ | ②Every week  ______ | ③Every Month  _____ | ④Every Year  _____ | ⑤Not at all  _____ |  |
| 42. leaf lettuce | ①Every day  ______ | ②Every week  ______ | ③Every Month  _____ | ④Every Year  _____ | ⑤Not at all  _____ |  |
| 43. coriander | ①Every day  ______ | ②Every week  ______ | ③Every Month  _____ | ④Every Year  _____ | ⑤Not at all  _____ |  |
| 44. crowndaisy chrysanthemum | ①Every day  ______ | ②Every week  ______ | ③Every Month  _____ | ④Every Year  _____ | ⑤Not at all  _____ |  |
| 45. baby Chinese cabbage | ①Every day  ______ | ②Every week  ______ | ③Every Month  _____ | ④Every Year  _____ | ⑤Not at all  _____ |  |
| 46. lettuce | ①Every day  ______ | ②Every week  ______ | ③Every Month  _____ | ④Every Year  _____ | ⑤Not at all  _____ |  |
| 47. mushroom | ①Every day  ______ | ②Every week  ______ | ③Every Month  _____ | ④Every Year  _____ | ⑤Not at all  _____ |  |
| 48. shii-take | ①Every day  ______ | ②Every week  ______ | ③Every Month  _____ | ④Every Year  _____ | ⑤Not at all  _____ |  |
| 49. black fungus | ①Every day  ______ | ②Every week  ______ | ③Every Month  _____ | ④Every Year  _____ | ⑤Not at all  _____ |  |
| 50. sea-tangle | ①Every day  ______ | ②Every week  ______ | ③Every Month  _____ | ④Every Year  _____ | ⑤Not at all  _____ |  |

(6) Fruits

| Names of food | | Frequency of food consumption | | | | | Amt. consumed  (50 g) |
| --- | --- | --- | --- | --- | --- | --- | --- |
| 51. apple | | ①Every day  ______ | ②Every week  ______ | ③Every Month  _____ | ④Every Year  _____ | ⑤Not at all  _____ |  |
| 52. pear | | ①Every day  ______ | ②Every week  ______ | ③Every Month  _____ | ④Every Year  _____ | ⑤Not at all  _____ |  |
| 53. pevery | | ①Every day  ______ | ②Every week  ______ | ③Every Month  _____ | ④Every Year  _____ | ⑤Not at all  _____ |  |
| 54. jujube | | ①Every day  ______ | ②Every week  ______ | ③Every Month  _____ | ④Every Year  _____ | ⑤Not at all  _____ |  |
| 55. winter jujube | | ①Every day  ______ | ②Every week  ______ | ③Every Month  _____ | ④Every Year  _____ | ⑤Not at all  _____ |  |
| 56. green grape | | ①Every day  ______ | ②Every week  ______ | ③Every Month  _____ | ④Every Year  _____ | ⑤Not at all  _____ |  |
| 57. red grape | | ①Every day  ______ | ②Every week  ______ | ③Every Month  _____ | ④Every Year  _____ | ⑤Not at all  _____ |  |
| 58. pomegranate | | ①Every day  ______ | ②Every week  ______ | ③Every Month  _____ | ④Every Year  _____ | ⑤Not at all  _____ |  |
| 59. persimmon | | ①Every day  ______ | ②Every week  ______ | ③Every Month  _____ | ④Every Year  _____ | ⑤Not at all  _____ |  |
| 60. strawberry | | ①Every day  ______ | ②Every week  ______ | ③Every Month  _____ | ④Every Year  _____ | ⑤Not at all  _____ |  |
| 61. actinidia chinensis | | ①Every day  ______ | ②Every week  ______ | ③Every Month  _____ | ④Every Year  _____ | ⑤Not at all  _____ |  |
| 62. orange | | ①Every day  ______ | ②Every week  ______ | ③Every Month  _____ | ④Every Year  _____ | ⑤Not at all  _____ |  |
| 63. citrus | | ①Every day  ______ | ②Every week  ______ | ③Every Month  _____ | ④Every Year  _____ | ⑤Not at all  _____ |  |
| 64. pomelo | | ①Every day  ______ | ②Every week  ______ | ③Every Month  _____ | ④Every Year  _____ | ⑤Not at all  _____ |  |
| 65. pineapple | | ①Every day  ______ | ②Every week  ______ | ③Every Month  _____ | ④Every Year  _____ | ⑤Not at all  _____ |  |
| 66. litchi | | ①Every day  ______ | ②Every week  ______ | ③Every Month  _____ | ④Every Year  _____ | ⑤Not at all  _____ |  |
| 67. mango | ①Every day  ______ | ②Every week  ______ | ③Every Month  _____ | ④Every Year  _____ | ⑤Not at all  _____ |  |  |
| 68. banana | ①Every day  ______ | ②Every week  ______ | ③Every Month  _____ | ④Every Year  _____ | ⑤Not at all  _____ |  |  |
| 69. papaya | ①Every day  ______ | ②Every week  ______ | ③Every Month  _____ | ④Every Year  _____ | ⑤Not at all  _____ |  |  |
| 70. pitaya | ①Every day  ______ | ②Every week  ______ | ③Every Month  _____ | ④Every Year  _____ | ⑤Not at all  _____ |  |  |
| 71. durian | ①Every day  ______ | ②Every week  ______ | ③Every Month  _____ | ④Every Year  _____ | ⑤Not at all  _____ |  |  |
| 72. watermelon | ①Every day  ______ | ②Every week  ______ | ③Every Month  _____ | ④Every Year  _____ | ⑤Not at all  _____ |  |  |
| 73. Hami melon | ①Every day  ______ | ②Every week  ______ | ③Every Month  _____ | ④Every Year  _____ | ⑤Not at all  _____ |  |  |
| 74. melon | ①Every day  ______ | ②Every week  ______ | ③Every Month  _____ | ④Every Year  _____ | ⑤Not at all  _____ |  |  |
| 75. mangosteen | ①Every day  ______ | ②Every week  ______ | ③Every Month  _____ | ④Every Year  _____ | ⑤Not at all  _____ |  |  |

(7) Livestock and its products

| Names of food | Frequency of food consumption | | | | | Amt. consumed  (50 g) |
| --- | --- | --- | --- | --- | --- | --- |
| 76. Pork | ①Every day  ______ | ②Every week  ______ | ③Every Month  _____ | ④Every Year  _____ | ⑤Not at all  _____ |  |
| 77. Pork liver | ①Every day  ______ | ②Every week  ______ | ③Every Month  _____ | ④Every Year  _____ | ⑤Not at all  _____ |  |
| 78. pork intestine | ①Every day  ______ | ②Every week  ______ | ③Every Month  _____ | ④Every Year  _____ | ⑤Not at all  _____ |  |
| 79. beef | ①Every day  ______ | ②Every week  ______ | ③Every Month  _____ | ④Every Year  _____ | ⑤Not at all  _____ |  |
| 80. beef liver | ①Every day  ______ | ②Every week  ______ | ③Every Month  _____ | ④Every Year  _____ | ⑤Not at all  _____ |  |
| 81. mutton | ①Every day  ______ | ②Every week  ______ | ③Every Month  _____ | ④Every Year  _____ | ⑤Not at all  _____ |  |
| 82. mutton liver | ①Every day  ______ | ②Every week  ______ | ③Every Month  _____ | ④Every Year  _____ | ⑤Not at all  _____ |  |
| 83. mutton intestine | ①Every day  ______ | ②Every week  ______ | ③Every Month  _____ | ④Every Year  _____ | ⑤Not at all  _____ |  |

(8) Poultry and its products

| Names of food | Frequency of food consumption | | | | | Amt. consumed  (50 g) |
| --- | --- | --- | --- | --- | --- | --- |
| 84. chicken | ①Every day  ______ | ②Every week  ______ | ③Every Month  _____ | ④Every Year  _____ | ⑤Not at all  _____ |  |
| 85. duck | ①Every day  ______ | ②Every week  ______ | ③Every Month  _____ | ④Every Year  _____ | ⑤Not at all  _____ |  |
| 86. goose | ①Every day  ______ | ②Every week  ______ | ③Every Month  _____ | ④Every Year  _____ | ⑤Not at all  _____ |  |

(9) Milk and its products

| Names of food | Frequency of food consumption | | | | | Amt. consumed  (50 g) |
| --- | --- | --- | --- | --- | --- | --- |
| 87. milk | ①Every day  ______ | ②Every week  ______ | ③Every Month  _____ | ④Every Year  _____ | ⑤Not at all  _____ |  |
| 88. yogurt | ①Every day  ______ | ②Every week  ______ | ③Every Month  _____ | ④Every Year  _____ | ⑤Not at all  _____ |  |
| 89. milk powder | ①Every day  ______ | ②Every week  ______ | ③Every Month  _____ | ④Every Year  _____ | ⑤Not at all  _____ |  |

(10)Eggs and its products

| Names of food | Frequency of food consumption | | | | | Amt. consumed  (50 g) |
| --- | --- | --- | --- | --- | --- | --- |
| 90. egg | ①Every day  ______ | ②Every week  ______ | ③Every Month  _____ | ④Every Year  _____ | ⑤Not at all  _____ |  |

(11) Sea food

| Names of food | Frequency of food consumption | | | | | Amt. consumed  (50 g) | Note |
| --- | --- | --- | --- | --- | --- | --- | --- |
| 91. carp | ①Every day  ______ | ②Every week  ______ | ③Every Month  _____ | ④Every Year  _____ | ⑤Not at all  _____ |  |  |
| 92. crucian | ①Every day  ______ | ②Every week  ______ | ③Every Month  _____ | ④Every Year  _____ | ⑤Not at all  _____ |  |  |
| 93.hairtail | ①Every day  ______ | ②Every week  ______ | ③Every Month  _____ | ④Every Year  _____ | ⑤Not at all  _____ |  |  |
| 94. yellow croaker | ①Every day  ______ | ②Every week  ______ | ③Every Month  _____ | ④Every Year  _____ | ⑤Not at all  _____ |  |  |
| 95. shrimp | ①Every day  ______ | ②Every week  ______ | ③Every Month  _____ | ④Every Year  _____ | ⑤Not at all  _____ |  |  |
| 96. others (please give the ‘name of the food ’in the ‘Note’ column) | ①Every day  ______ | ②Every week  ______ | ③Every Month  _____ | ④Every Year  _____ | ⑤Not at all  _____ |  |  |

(12) Snack

| Names of food | Frequency of food consumption | | | | | Amt. consumed  (50 g) |
| --- | --- | --- | --- | --- | --- | --- |
| 97. Sugar-sweetened preserved fruits | ①Every day  ______ | ②Every week  ______ | ③Every Month  _____ | ④Every Year  _____ | ⑤Not at all  _____ |  |
| 98. biscuit | ①Every day  ______ | ②Every week  ______ | ③Every Month  _____ | ④Every Year  _____ | ⑤Not at all  _____ |  |
| 99. fried chips | ①Every day  ______ | ②Every week  ______ | ③Every Month  _____ | ④Every Year  _____ | ⑤Not at all  _____ |  |
| 100. chocolate | ①Every day  ______ | ②Every week  ______ | ③Every Month  _____ | ④Every Year  _____ | ⑤Not at all  _____ |  |
| 101. other sweets | ①Every day  ______ | ②Every week  ______ | ③Every Month  _____ | ④Every Year  _____ | ⑤Not at all  _____ |  |

(13) Beverage

| Names of food | Frequency of food consumption | | | | | Amt. consumed  (50 g) |
| --- | --- | --- | --- | --- | --- | --- |
| 102. sugar-sweetened drink | ①Every day  ______ | ②Every week  ______ | ③Every Month  _____ | ④Every Year  _____ | ⑤Not at all  _____ |  |

(14) Ice cream

| Names of food | Frequency of food consumption | | | | | Amt. consumed  (50 g) |
| --- | --- | --- | --- | --- | --- | --- |
| 103. ice cream (84g/per standard size icecream) | ①Every day  ______ | ②Every week  ______ | ③Every Month  _____ | ④Every Year  _____ | ⑤Not at all  _____ |  |

**PART THREE LIFESTYLE AND PHSICAL CONDITION**

**Ⅰ.Calcium deficiency and supplementation**

1. Have you ever been diagnosed with [rickets](app:ds:rickets) when you were aged 0-3 years old?

①yes   ②no ③unknown

2. Have you ever been diagnosed with bone deformity when you were aged 0-3 years old?

①X-shaped leg  ②O-shaped leg   ③pigeon chest   ④beaded chest ⑤no

3. Have you ever had other symptoms of calcium deficiency or vitamin D deficiency when you were aged 0-3 years old?

①dentation retardation ②night crying ③no ④unknown

4. Have you ever taken calcium supplementation, or vitamin D/ cod liver oil during you were 0-15 years old？

①yes   ②no ③unknown

**Ⅱ. Prior disease history**

1. Have you ever been diagnosed with the following diseases?

| Name of diseases | A. Whether or not it has been diagnosed | B. When have you been diagnosed? | C. Did you control the disease? | D. What kind of method have you performed to control the disease? |
| --- | --- | --- | --- | --- |
| 1.type 2 diabetes | 1. yes 2. no | _____year  ____ month | 1. yes 2. no | ①taking medicine   ②insulin injection ③diet control ④moderate exercise ⑤glucose monitoring  ⑥others ____________________ |
| 2.hypertension | 1. yes 2. no | _____year  ____ month | 1. yes 2. no | ①taking medicine in following doctor’s advice   ②taking medicine when having symptom ③diet control ④moderate exercise ⑤ blood pressure monitoring ⑥others ______ |
| 3. hyperlipidemia | ① yes  ② no | _____year  ____ month | ① yes  ② no | ①taking medicine in following doctor’s advice   ②diet control ③moderate exercise ④blood lipid monitoring ⑤others __________________ |

1. Have you ever been diagnosed with the following diseases?

| Name of diseases | A. Whether or not it has been diagnosed | B. When have you been diagnosed? |
| --- | --- | --- |
| 4. coronary heart disease | ①yes   ②no | _____year ____ month |
| 5. stroke | ①yes   ②no | _____year ____ month |
| 6. nephropathy (such as nephritis, kidney calculi, etc.) | ①yes   ②no | _____year ____ month |
| 7. liver disease (such as fatty liver, hepatitis, etc.) | ①yes   ②no | _____year ____ month |
| 8. blood system disease | ①yes   ②no | _____year ____ month |
| 9. chronic obstructive pulmonary disease | ①yes   ②no | _____year ____ month |
| 10. asthma | ①yes   ②no | _____year ____ month |
| 11.other chronic disease | ①yes (name of the disease_____)  ②no | _____year ____ month |

**Ⅲ. Family history disease**

Among these first degree relatives including your grandparents, parents, sisters, and brothers, has anybody ever been diagnosed with the following diseases?

| Name of diseases | A. Whether or not it has been diagnosed | B. What is the relationship with you and how old was he or she diagnosed? |
| --- | --- | --- |
| 1. type 2 diabetes | ①yes   ②no | relationship_____ ,_______ years old |
| 2. obesity | ①yes   ②no | relationship_____ ,_______ years old |
| 3. hypertension | ①yes   ②no | relationship_____ ,_______ years old |
| 4. hyperlipidemia | ①yes   ②no | relationship_____ ,_______ years old |
| 5. coronary heart disease | ①yes   ②no | relationship_____ ,_______ years old |
| 6. cerebrovascular disease, such as cerebral hemorrhage, subarachnoid hemorrhage, cerebral thrombosis, cerebral embolism, etc. | ①yes   ②no | relationship_____ ,_______ years old |
| 7. chronic respiratory disease, such as chronic bronchitis, emphysema, asthma | ①yes   ②no | relationship_____ ,_______ years old |

**Ⅳ. Have you ever been in the following situation in the past year?**

| Situation | A. Whether or not you have been in the situation |
| --- | --- |
| 1. taking or inject medicine or health products containing vitamin D or calcium | ①yes, the name of the medicine or health product_______, dose_______  ②no |
| 2. taking sugar-reducing medicine or assisting sugar-reducing health product | ①yes   ②no |
| 3. taking weight-reducing medicine or assisting weight -reducing health product | ①yes   ②no |
| 4. on a diet for weight loss | ①yes   ②no |
| 5. taking medicine for weight loss | ①yes   ②no |
| 6. going to gym for weight loss | ①yes   ②no |
| 7. square dance for weight loss | ①yes   ②no |
| 8. having been diagnosed with bone fracture, hyperthyroidism, hypothyroidism, or hypercalcemia | ①yes   ②no |
| 9. taking hormone replacement therapy | ①yes   ②no |
| 10.taking medicine for treating osteoporosis, such as calcitonin or diphosphonate | ①yes   ②no |

**Ⅴ. Labor intensity in the past year**

Which kinds of labor intensity do you belong to?

①light (including retire person, office staff, watch mender, shop assistant, technician in lab, teveryers, and etc.)

②medium (including student, driver, electrician, and etc.)

③heavy( including farmers, dancer, athlete, stevedore, timberjack, miner, manson, and etc.)

**Ⅵ. Exercise in the past year**

Regular exercise was defined as any kind of recreational or sport physical activity other than walking for work or life performed three or more days per week for at least 30 minutes.

1.Did you take regular exercise in the past year?

①yes(if yes, please answer the following question 2 and 3)

②no

2.What kind of exercise did you take? _______

3. How often did you take regular exercise? ______ times/week

4. How long did you take regular exercise each time? _____hours _____ minutes

**Ⅶ. Smoking habit** **in the past year**

1. Have you ever smoked at least 100 cigarettes lifetime or smoke every day or some days now?

①yes (if yes, please answer the following two questions)

②no

2. How old were you when you started to smoke? _____ years old

3. How many cigarettes did you smoke? _______ cigarettes/day

4. How long have you smoked? _______years ______months

5. How many days in a week were you often endured in passive smoking more than fifteen minutes per day?

①almost no ②yes, ______days

6. Have you ever smoked but now quit smoking?

①yes (if yes, please answer the following question)

②no

7. How many years have you quitted smoking? _________years _____months ____days

**Ⅷ. Drinking habit in the past year**

1. Have you drunk alcohol often? (alcohol including liquor, beer, grape wine, rice wine and highland barley wine)?

①yes (if yes, please answer the following questions)

②no

| Type of alcohol | Frequency  (times/month) | Amount each time | Notes |
| --- | --- | --- | --- |
| 2. liquor | ______ | ____ ml | Degree:①＜42 ②≥42 |
| 3.beer | ______ | ____ml | / |
| 4.grape wine | ______ | ____ ml | / |
| 5.rice wine | ______ | ____ ml | / |
| 6. highland barley wine | ______ | ____ ml | / |

7. How long have you drunk? _______years ____ months

**Ⅸ. Sleep** **habit in the past year**

1.What time do you usually go to bed at night ? _____ pm

2.How long do you take from going to bed to fall asleep? _____minutes

3.What time do you usually get up in the morning? _____ am

4.How many hours do you usually sleep every night? ______ hours (do not including in-bed time)

5. Nocturnal sleep

| Phenomena | Frequency |
| --- | --- |
| A. difficulty in falling asleep | ①never ②< once/week ③1-2 times/week ④≥ 3 times/week |
| B. easily awake or awake early | ①never ②< once/week ③1-2 times/week ④≥ 3 times/week |
| C. go to toilet | ①never ②< once/week ③1-2 times/week ④≥ 3 times/week |
| D. poor breath | ①never ②< once/week ③1-2 times/week ④≥ 3 times/week |
| E. cough and snore | ①never ②< once/week ③1-2 times/week ④≥ 3 times/week |
| F. feel cold | ①never ②< once/week ③1-2 times/week ④≥ 3 times/week |
| G. feel hot | ①never ②< once/week ③1-2 times/week ④≥ 3 times/week |
| H. nightmare | ①never ②< once/week ③1-2 times/week ④≥ 3 times/week |
| I. pain and discomfort | ①never ②< once/week ③1-2 times/week ④≥ 3 times/week |
| J. Others _________ | ①never ②< once/week ③1-2 times/week ④≥ 3 times/week |

6. In general, how do you feel your sleep quality?

①very good ②good ③poor ④very poor

7. How often do you take medicine for hypnosis?

①never ②< once/week ③1-2 times/week ④≥ 3 times/week

8. How often do you feel sleepiness?

①never ②< once/week ③1-2 times/week ④≥ 3 times/week

9. How often do you feel anenergia when working?

①never ②occasional ③sometimes ④often

10. A. How often do you take a nap in the day time?

①never ②< once/week ③1-2 times/week ④≥ 3 times/week

B. How long do you have the nap? ______ hours _____minutes

**PART FOUR BODY MEASUREMENT**

This part is to measure the height, weight, waistline, body fat percentage, and blood pressure of the interviewees. To ensure the accuracy of the measurements, the interviewees are required to wear only one layer of clothes. If this is refused, estimate the actual values and record the clothes the interviewees wore in the remark columns.

| Measured body site | The first measurement | The second measurement | The third measurement |
| --- | --- | --- | --- |
| 1.height (cm) | ________ | ________ | ________ |
| 2.weight (kilogram) | ________ | ________ | ________ |
| 3. waistline (cm) | ________ | ________ | ________ |
| 4.body fat percentage | ________ | ________ | ________ |
| 5. systolic blood pressure(mmHg) | ________ | ________ | ________ |
| 6. diastolic blood pressure(mmHg) | ________ | ________ | ________ |

**INTERVIEWER POSTSCRIPT**

1. The result of the survey: 1. …. Completion

2. …. Partly completion

3. …. Refuse participation

88…..others ___________

2. Signature of the interviewer:   Date: _____________

3. Signature of the interviewee: Date: _____________

4. Signature of the quality controller: Date: _____________
